# ‘Optimising’ breastfeeding: what can we learn from evolutionary, comparative and anthropological aspects of lactation?

**DOI:** 10.1186/s12916-019-1473-8

**Published:** 2020-01-09

**Authors:** Mary S. Fewtrell, Nurul H. Mohd Shukri, Jonathan C. K. Wells

**Affiliations:** 1 Childhood Nutrition Research Centre, UCL Great Ormond Street Institute of Child Health, 30 Guilford Street, London, WC1N 1EH UK; 2 Department of Nutrition & Dietetics, Faculty of Medicine & Health Sciences, Universiti Putra Malaysia, Selangor, Malaysia

**Keywords:** Breastfeeding, Lactation, Primate, Human, Anthropology, Evolution, Mother–infant conflict, Learning, Signalling

## Abstract

**Background:**

Promoting breastfeeding is an important public health intervention, with benefits for infants and mothers. Even modest increases in prevalence and duration may yield considerable economic savings. However, despite many initiatives, compliance with recommendations is poor in most settings – particularly for exclusive breastfeeding. Mothers commonly consult health professionals for infant feeding and behavioural problems.

**Main body:**

We argue that broader consideration of lactation, incorporating evolutionary, comparative and anthropological aspects, could provide new insights into breastfeeding practices and problems, enhance research and ultimately help to develop novel approaches to improve initiation and maintenance. Our current focus on breastfeeding as a strategy to improve health outcomes must engage with the evolution of lactation as a flexible trait under selective pressure to maximise reproductive fitness. Poor understanding of the dynamic nature of breastfeeding may partly explain why some women are unwilling or unable to follow recommendations.

**Conclusions:**

We identify three key implications for health professionals, researchers and policymakers. Firstly, breastfeeding is an adaptive process during which, as in other mammals, variability allows adaptation to ecological circumstances and reflects mothers’ phenotypic variability. Since these factors vary within and between humans, the likelihood that a ‘one size fits all’ approach will be appropriate for all mother-infant dyads is counterintuitive; flexibility is expected. From an anthropological perspective, lactation is a period of tension between mother and offspring due to genetic ‘conflicts of interest’. This may underlie common breastfeeding ‘problems’ including perceived milk insufficiency and problematic infant crying. Understanding this – and adopting a more flexible, individualised approach – may allow a more creative approach to solving these problems. Incorporating evolutionary concepts may enhance research investigating mother–infant signalling during breastfeeding; where possible, studies should be experimental to allow identification of causal effects and mechanisms. Finally, the importance of learned behaviour, social and cultural aspects of primate (especially human) lactation may partly explain why, in cultures where breastfeeding has lost cultural primacy, promotion starting in pregnancy may be ineffective. In such settings, educating children and young adults may be important to raise awareness and provide learning opportunities that may be essential in our species, as in other primates.

## Background

Promoting and supporting breastfeeding is an important public health intervention, with multiple health benefits for infants and mothers [1] and the potential for considerably reduced healthcare costs from even modest increases in prevalence and duration [2, 3]. The medical and public health perspective is that milk is primarily a source of nutrition and lactation is a largely one-way process in which the mother provides whatever her infant needs. This results in prescriptive recommendations for breastfeeding based on nutritional considerations, combined – in more recent years – with evidence for health effects of infant feeding practices. The World Health Organisation (WHO) recommends that mothers should exclusively breastfeed their infant for 6 months, followed by continued breastfeeding alongside complementary feeding for 2 years [4]. However, despite numerous initiatives to improve breastfeeding initiation and duration over many years, compliance with recommendations is poor in most settings. Breastfeeding initiation and duration is particularly low in some Western countries, including the UK [1]. Furthermore, infant feeding and behaviour problems are frequent causes for consultation with a health professional.

Here, we consider lactation from a broader perspective, including its evolution, variability between mammals (including non-human primates) and its role in ‘signalling’ between mother and offspring under the selective pressure to maximise reproductive fitness. We discuss how this alternative perspective could provide new insights into breastfeeding practices and problems, enhance research and assist in the development of novel approaches to improve breastfeeding initiation and maintenance.

### Evolution of lactation

Lactation is the current manifestation of an evolutionary process with an origin that long pre-dates the emergence of mammals, live birth and placentation [5, 6]. It is generally accepted that precursors of lactation originated in our pre-mammalian ancestors more than 250 million years ago – primarily as a source of fluid to prevent eggs from drying out. The presence of antimicrobial factors in milk is proposed to have emerged through an early adaptation to prevent the eggs and maternal skin from becoming infected in warm, moist environments. It is thought that the mammary gland originated via adaptation of apocrine sweat glands. Indeed, the two share several similarities, including secretion by both exocytosis and budding and association with myoepithelial cells and hair follicles. However, mammary glands have several further specialised adaptations, including their ability to undergo repeated bouts of proliferation and secretion followed by involution, a greater variety of secretions and more complex hormonal control.

In the earliest evolutionary stages, it is thought that a significant nutritional role for such secretions was secondary; and many nutritional components appear to have developed from prior immune or antimicrobial functions [6]. For example, the milk protein alpha-lactalbumin, which regulates the production of lactose, probably originated from the antimicrobial enzyme lysozyme and was likely to have been involved in oligosaccharide synthesis before assuming a nutritional role. Several components of the milk fat globule membrane – one of the most conserved components of mammalian milk – also, initially, had immune functions. Genes related to the mammary gland and lactation are also more highly conserved than those for other somatic functions [7]; and those coding for proteins involved in secretory functions, such as the milk fat globule membrane, are most highly conserved.

#### Advantages of nutritional fluids

The provision of nutritional fluids for the offspring confers several potential advantages in the context of maternal care. It allows the mother to feed her offspring relatively independently of the source of her own food, relying on her stores of energy, macronutrients and micronutrients [8]. It also allows offspring to reach a greater size or degree of maturity before acquiring the physiological and anatomical characteristics needed to consume a specialised diet. A reduced reliance on the egg for nutrition over evolutionary timescales also allowed a reduction in the size of the egg. This shift in reproductive strategy is not confined to placental nutrition and lactation in mammals; some birds (e.g. pigeons) provide crop milk for their chicks. This differs from lactation, however, since the fluid is passed directly from mouth to mouth and is produced from holocrine glands, where whole cells slough off into the fluid [6].

**Fig. 1:**
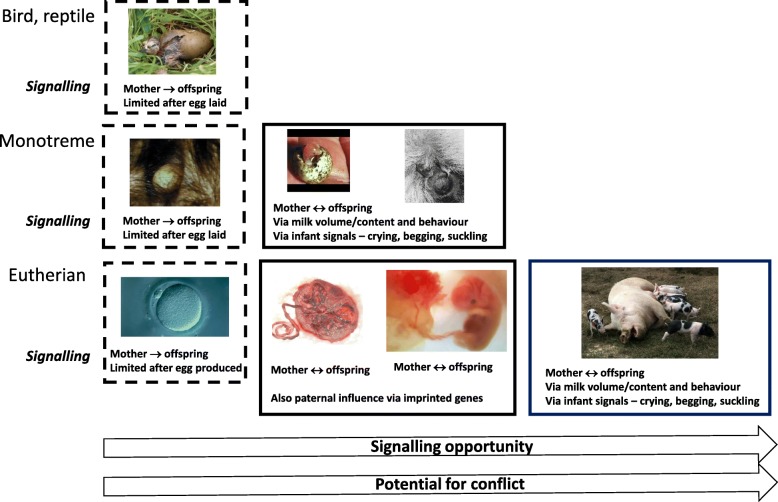
Potential signalling routes between mother and offspring. These routes illustrate the different contributions made by the egg, placenta, amniotic fluid and lactation and their effects on both signalling opportunity and potential for mother–offspring conflict

The provision of nutritional fluids has another important benefit beyond the provision of fluid, antimicrobial factors and nutrients. It provides enhanced and extended opportunities for signalling or communication between mother and offspring beyond those possible via an egg (illustrated in Fig. 1), where the opportunity for signalling is negligible between laying and hatching. Lactation offers a prolonged period, following placental nutrition, during which the mother can continue to buffer her offspring against external ecological stresses, while drawing on her own nutritional reserves to support infant development [9]. This greater opportunity for bidirectional signalling between mother and offspring has the advantage of greater flexibility and responsiveness, but it also increases the period during which physiological ‘conflicts of interest’ between mother and offspring can occur [10, 11].

### Parent–offspring conflict during lactation

Parent–offspring conflict theory [11] proposes that, to optimise its own reproductive fitness, each offspring will demand more resources from the mother than the level that would maximise maternal fitness. The mother is equally related to all her offspring and, all other things being equal, maximises fitness by investing equally in them, whereas each offspring gains to a lesser degree than the mother from the fitness of its siblings [11]. Contrasting selective pressures that act on paternal and maternal genes in the offspring then enhance this ‘tug of war’ [12]. The resulting conflict of interest over the magnitude of maternal investment is predicted to peak during periods that are energetically costly, and lactation is well established to have greater metabolic costs than pregnancy [13, 14]. All other things being equal, the mother maximises her fitness by weaning the offspring earlier, regaining her fertility and producing the next offspring, whereas the offspring maximises fitness by prolonging lactation and maternal investment. Thus lactation should not be regarded as a one-way process in which the mother has her offspring’s best interests at heart, but as a two way ‘negotiation’ over maternal resources [15]. Tension is expected, and may plausibly underlie some commonly encountered problems related to breastfeeding and infant crying. Ironically, crying is a costly process that, in breastfed infants, must ultimately be funded by maternal metabolism [15].

A further consideration is variability in the partitioning of maternal investment between pregnancy and lactation, which may reflect the mother’s own life history strategy and have consequences for the offspring’s growth pattern and later health [16]. In this context, any information conveyed by milk transfer does not relate directly to the external environment, but rather to the current maternal phenotype and her prior developmental trajectory.

It is through signalling between mother and offspring that parent–offspring conflict can be resolved, taking into account the phenotype of each party. This means that variability in lactation is expected – even for mothers and offspring experiencing identical current environments – on account of variability in maternal and infant phenotype that in each generation relates to contrasting developmental experience. For example, a baby born with low birthweight may undergo catch-up growth during the early postnatal period [17]. This, in turn, may affect the quantity and regulation of milk transfer.

### Lactation strategies

Milk has allowed mammals to develop various reproductive and lactation strategies, resulting in great variation in the duration and frequency of milk feeding, as well as in the composition of milk. Under the selective pressure to maximise reproductive fitness, lactation has evolved to be flexible and responsive to multiple components of maternal phenotypic variability; hence, the existence of a single optimal duration of lactation – even within a single species – may be considered biologically implausible.

Primates generally have slow-growing offspring that are dependent on their parents for long periods [18]. The daily nutrient transfer from mothers to offspring by milk is relatively low compared to many other mammals: this has the advantage of spreading the energy cost of investment over a longer period [19], but also the disadvantage of extending the period during which conflicts of interest can occur between mother and offspring.

Primate lactation is also characterised by a lesser role of instinct relative to most other mammals and a greater role of learning and social aspects of infant care [20, 21]. This has the advantage of greater flexibility to adapt to different environments or circumstances, but – paradoxically – results in vulnerability if appropriate early learning experiences are not available. This was observed when the first chimpanzees and gorillas were born in captivity: their mothers showed no awareness of the need to feed their infant, having been deprived of the normal experience of observing relatives feeding and caring for their offspring [22–24].

Human lactation is characterised by further adaptations. Relative to other primates, human milk is rich in human milk oligosaccharides [25]. It has been suggested that this adaptation facilitated the increased population density characteristic of sedentary communities by enhancing defence against infections [26]. Humans also have shorter periods of lactation and shorter interbirth intervals than other apes [27]. This may have been facilitated by the development of a complementary feeding period, during which the offspring simultaneously receives nutrition from milk and from other foods provided by the mother. This complementary feeding period is not observed in most other primates [28, 29], although in callitrichid primates (marmosets and tamarins), who also have relatively short periods of lactation, the infant is carried by the father and is provisioned with foods by group members for about half of the lactation period [30]. By shortening the period of lactational amenorrhoea, complementary feeding allows the mother to reproduce again sooner. However, such a strategy is also predicted to be flexible, since providing non-breastmilk foods early during development would only be advantageous under conditions that allowed the weaned infant to survive and thrive.

In the past, the provision of complementary foods would have required premastication by the mother or carer. However, in recent times – and in most settings – cooking or food processing has displaced this behavioural adaptation and allowed even earlier introduction of complementary foods [31]. This may be advantageous in terms of the mother’s reproductive fitness and may, for example, have played a key role in population growth following the origins of agriculture [32]. However, there could also be costs if the focus is on health outcomes rather than reproductive fitness.

It can be hypothesised that, in optimal environmental conditions, critical windows for signalling would be shorter since there is less need for the mother to communicate information about a stable environment and less tension over resources. By contrast, in unstable or suboptimal conditions, it would be advantageous to prolong the period of signalling and parent–offspring conflict might be exacerbated [33]. While evidence supporting this hypothesis in humans is limited, primates have been observed to wean earlier when resources are plentiful [34]. Conversely, primiparous rhesus macaque mothers were observed to return to oestrus later than multiparous mothers, probably because their lower bodyweight reduced milk yield, which, in turn, stimulated higher infant suckling rates [35]. These observations further reinforce the concept of lactation as a process that has evolved to be flexible and highlight the potential difficulties of implementing recommendations that specify fixed optimal periods of lactation [21].

Although primates do not generally have a complementary feeding period during which the infant receives nutrition from milk and solid foods provided by the mother, infant apes may consume small, nutritionally insignificant amounts of foods dropped by or taken from the mother, or provided by other members of the group [26]. Exposure to food allergens or tastes and flavours in this way could potentially assist the development of tolerance to food antigens or locally relevant taste and food preferences – two issues that are currently of great research interest in human infants [36]. It may therefore be considered that complementary feeding in humans has two distinct components: nutritional and non-nutritional, with the latter including both behavioural and immunological aspects.

Compared with lactation in other primates, human lactation exhibits an even greater reliance on learning. Lactation, along with other aspects of childbirth, also has considerable social and cultural importance, as illustrated by the large number of rituals around childbirth and lactation. In many societies, mothers are given lactagogues and herbal medicines around the time of birth to stimulate milk production. The mother and new child are also often secluded [37] – a behaviour that arose to ward off the threat of evil during this vulnerable time, but which now has implications for social support provided during early breastfeeding. Other cultural components of breastfeeding include links between weaning and other elements of the infant’s socialisation. A poor understanding of the way in which knowledge of lactation is embedded in cultural and traditional practices may help to explain difficulties in promoting breastfeeding among pregnant women and new mothers in industrialised societies, where breastfeeding is losing its cultural primacy. Many children and young people now grow up without the necessary exposure and learning opportunities and are losing access to the informal social networks that traditionally facilitated transmission of the behaviour across generations [21]. Primiparous women in situations with limited family support may be particularly vulnerable. In some societies, the partner may play an important role in facilitating breastfeeding, emphasising the need to focus on education in both genders [38].

### Mechanisms of mother–offspring signalling

Dynamic interactions between mother and offspring are enabled by multiple forms of signalling, which can occur – for example – via the manipulation of egg phenotype, placental interactions, or during the postnatal period. The importance of each of these options varies between species. In mammals, postnatal signalling via lactation could affect feeding behaviour and milk transfer as well as milk composition. One intriguing example of behavioural signals that express the conflict of interest between mother and offspring concerns night-time suckling [39]. By studying the sleep-related phenotypes of infants with Prader–Willi and Angelman syndromes, Haig suggested that imprinted genes of paternal origin promote greater night-time waking, whereas imprinted genes of maternal origin favour less. These observations are consistent with the hypothesis that waking at night to suckle is an infant strategy to extend the mother’s lactational amenorrhea, thus delaying the birth of a younger sibling, securing the infant’s own breastmilk supply and enhancing its survival.

**Fig. 2:**
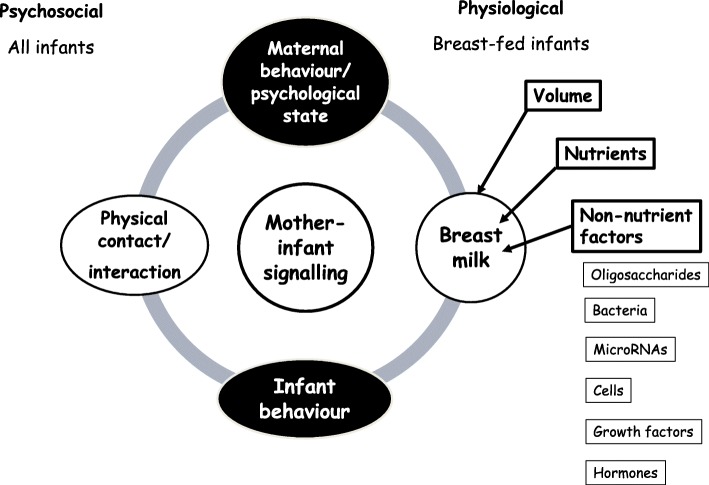
Potential postnatal signalling routes and mechanisms, including physiological and behavioural factors

In humans, signalling could occur via behavioural or psychological mechanisms, regardless of infant feeding mode (illustrated in Fig. 2), but physiological signalling could only occur via milk. Milk contains numerous possible signalling components, including nutrients, growth factors, hormones, bacteria, cells and microRNA. However, they are generally poorly understood, partly because of complex interrelationships that are difficult to disentangle using observational studies, but also reflecting methodological issues related to the schedule and sampling strategy used to obtain milk samples.

**Fig. 3:**
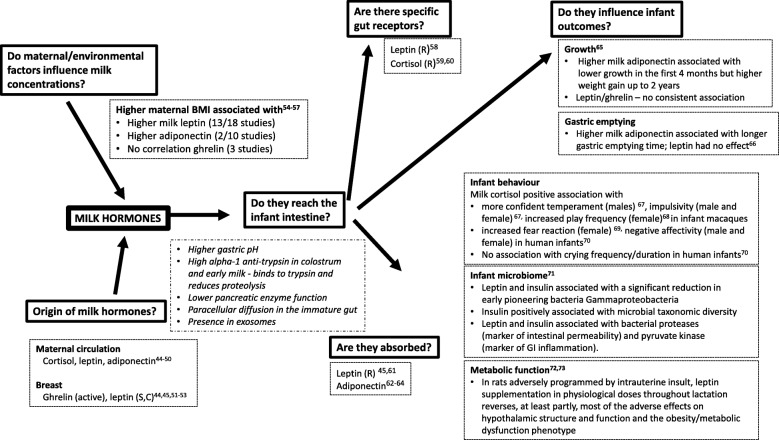
Evaluation of the plausibility of milk hormones as signals between mother and offspring. To determine whether or not a hormone acts as a signal between mother and offspring, we suggest it is important to establish its source (whether from maternal blood or synthesised in the breast); whether milk concentrations are influenced by maternal/environmental factors; that milk hormones can reach the infant intestine and (probably) be absorbed; and that milk hormones influence infant outcomes. The figure summarises published data for each of these steps for leptin, adiponectin, ghrelin and cortisol and highlights the relative lack of consistent data for all stages of the pathway; most studies examine one or two components and few have examined infant outcomes beyond growth and/or adiposity. Furthermore, all studies are observational, precluding decisions on causality. R: rodent; S: sheep; C: cow

Perhaps most importantly, although these components are proposed as signals between mother and infant, few studies have systematically investigated whether they fulfil key aspects of a potential ‘signal’; for example, whether their concentrations in milk are influenced by maternal or environmental factors or influence infant outcomes. These issues, described many years ago [40], are well illustrated by considering the case for milk hormones as potential signals (see Fig. 3 [44–74]). While there is some evidence to support each of the individual steps, few studies have simultaneously investigated more than two components, making it difficult to draw conclusions. Furthermore, the studies are observational, which precludes determination of causality.

## Conclusions

### Implications for health professionals, researchers and policymakers

**Fig. 4:**
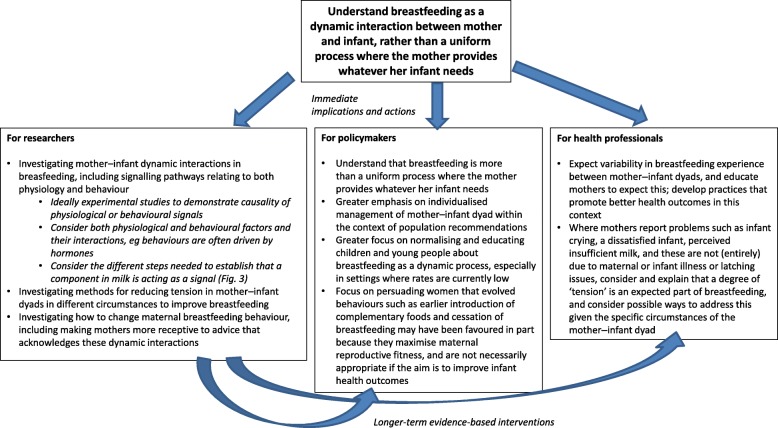
Suggested key action points for health professionals and policymakers and suggested future research directions. The central concept is lactation as a dynamic, flexible process, which is expected to differ between mother–infant dyads. Immediate implications and actions arising from this are suggested for health professionals and policymakers, together with suggested research directions. Research should provide evidence-based strategies for longer-term use by health professionals and policymakers

We have identified several areas in which a broader consideration of lactation could inform medical and public health views of lactation. Crucially, we should remember that although contemporary thinking on breastfeeding prioritises health and quality of life, selection favours traits that maximise inclusive fitness rather than health. Breastfeeding might appear to be simultaneously ‘natural’ and ‘optimal’ for health, but – in fact – interventions on breastfeeding intended to promote health must inevitably engage with a dynamic process characterised by multiple components of variability. Current approaches focus on whether and for how long women breastfeed. Based on the arguments presented here, we suggest a greater focus on improving *how* women breastfeed, so as to promote maternal and child health and wellbeing. We identify three key areas in which a broader perspective might contribute to a better understanding of breastfeeding problems and of why mothers do not seem willing or able to follow recommendations. Key points for policymakers, health professionals and researchers are provided in Fig. 4.

### Flexibility

Medical and public health recommendations on infant feeding focus on identifying the optimal biological ‘norm’ for breastfeeding to provide the infant with optimal nutrition, while at the same time minimising morbidity and mortality and improving health outcomes. However, the formulation of rigid guidelines for breastfeeding conflicts with the concept of breastfeeding as an adaptive process where, as already demonstrated both in humans and in other mammalian species, variability allows adaptation to local ecological circumstances and reflects the life history strategy of the mother. Since physiology and ecological environments vary between human populations, the likelihood that a one-size-fits-all approach will work for all mother–infant dyads is counterintuitive. Infants are not passive recipients of ‘optimal nutrition’ and mothers are not passive providers [21]. Understanding this, and expecting individual variation around what is regarded as ‘optimal’ in terms of nutrition and health outcomes, may improve maternal experiences and, by reducing stress associated with pressure to adhere to a fixed recommendation, may in itself improve breastfeeding outcomes.

### Tension is to be expected

From an evolutionary perspective, lactation is expected to be a period of tension deriving from a metabolic ‘conflict of interest’ between mother and offspring. This may underlie several commonly reported breastfeeding ‘problems’. For example, the observation that breastfeeding infants have a more challenging temperament than formula-fed infants [41] is to be expected rather than regarded as indicating abnormality. Mothers’ perceptions that they have insufficient milk or that their infant is not satisfied, and problematic crying and night-time suckling, may be other manifestations of mother–infant conflict [15, 39].

Greater acknowledgement and maternal awareness of this expected tension may provide a different perspective on some common infant feeding issues and – perhaps – more creative approaches to solving them. If a mother understands that such problems do not necessarily indicate a problem with the infant, or that she is doing something wrong, this in itself may reduce stress and anxiety. This may be particularly relevant in groups of mothers and infants for whom breastfeeding is more challenging and tension may be higher – including, for example, first-born infants, those born late preterm or early term, those with increased nutritional requirements for catch-up growth and situations in which maternal stress may cause the mother to divert energy away from lactation, resulting in a dissatisfied infant who demands more milk.

Considering breastfeeding practices and milk components as signals between mother and infant may improve approaches to breastfeeding support, but also enhance study design. However, given complex interrelationships between components, observational studies may not be particularly informative; experimental studies (randomised trials) are preferable to establish causal relationships and identify components that mediate observed effects.

Incorporating evolutionary concepts may enhance the design of such studies. Similarly, the use of experimental approaches in anthropological research on lactation may allow more robust conclusions to be drawn. For example, in a randomised trial combining clinical and anthropological concepts, the use of a simple relaxation intervention to reduce stress in breastfeeding mothers resulted in longer infant sleep duration and higher infant weight and body mass index (BMI) gain [42, 43]. The mean standardised body mass index (BMI SDS) scores of the intervention group showed a close match with the optimal growth of breastfed infants according to WHO growth standards, suggesting that the relaxation intervention allowed the infants to come closer to the ‘ideal’ growth pattern. However, long-term studies investigating health and functional outcomes beyond infancy are needed to define optimal growth patterns in different settings. The study findings were consistent with the hypothesis that reducing tension between mother and infant during lactation resulted in greater maternal investment in the infant. The trial also identified potential signalling mechanisms for investigation in future and larger studies. This intervention could readily be applied in other settings to improve breastfeeding outcomes and to further test biological and evolutionary hypotheses, for example, in groups of mothers and infants for whom greater tension is anticipated during lactation, such as following delivery of a preterm or low birthweight infant, or in situations of social or environmental stress. However, research is required to identify appropriate interventions for different settings and groups of mothers and infants.

### The importance of learning

Beyond evolutionary issues, an anthropological approach can also improve our understanding of the social and cultural basis of variability in lactation, which – again – may improve the success of interventions. For example, greater emphasis on the importance of learned behaviour and the social and cultural aspects in primate – and especially human – lactation, may help to explain why, in industrialised populations in which breastfeeding is no longer the norm, measures to promote breastfeeding that are directed predominantly at pregnant or postpartum mothers may not be very effective. In such settings, educating children and young adults – of both genders – may be important to raise awareness and provide the learning opportunities that may be essential in our species, as in other primates. A further consideration is how to convince women that behaviours related to lactation and complementary feeding, which evolved under the selective pressure to maximise reproductive fitness, may not necessarily be appropriate if the focus is entirely on improving health outcomes.

## Data Availability

Not applicable.
